# Pneumothorax spontané simultanément bilateral

**DOI:** 10.11604/pamj.2016.24.57.8729

**Published:** 2016-05-13

**Authors:** Hicham Souhi, Hanane El Ouazzani

**Affiliations:** 1 Service de Pneumologie de l'Hôpital Militaire d'Instruction Mohammed V, Rabat, Maroc

**Keywords:** Pneumothorax, spontané, bilatéral, Pneumothorax, spontaneous, bilateral

## Image en medicine

Le pneumothorax est un épanchement gazeux entre la plèvre viscérale et la plèvre pariétale. Le pneumothorax spontané est exceptionnellement bilatéral d'où la particularité de cette observation. Il s'agit d'un patient âgé de 47 ans, fumeur à 35 paquet/année, bronchitique chronique depuis 8 ans, admis aux urgences dans un tableau de douleur thoracique diffuse, polypnée, tirage intercostal et sus sternal, et cyanose des extrémités. Les diagnostics les plus probables étaient: une exacérbation de BPCO, une embolie pulmonaire ou un pneumothorax. L'examen pleuro pulmonaire trouve un syndrome d’épanchement aérique bilatéral. La radiographie thoracique montre une hyperclareté avasculaire bilatérale accentuée à gauche. Le scanner thoracique révèle un pneumothorax bilatéral plus important à gauche, sur poumon multibulleux. Le patient a bénéficié de la mise en place d'un drain pleural sous aspiration douce (-20 cmH2O) du côté gauche le plus décollé et une exsuflation du côté controlatéral, ainsi qu'un traitement symptomatique à base de corticothérapie par voie veineuse (Solumedrol) et nébulisation de salbutamol, couverture antibiotique et repos strict au lit. L’évolution a été marquée par le retour total du poumon à la paroi des 2 côtés.

**Figure 1:**
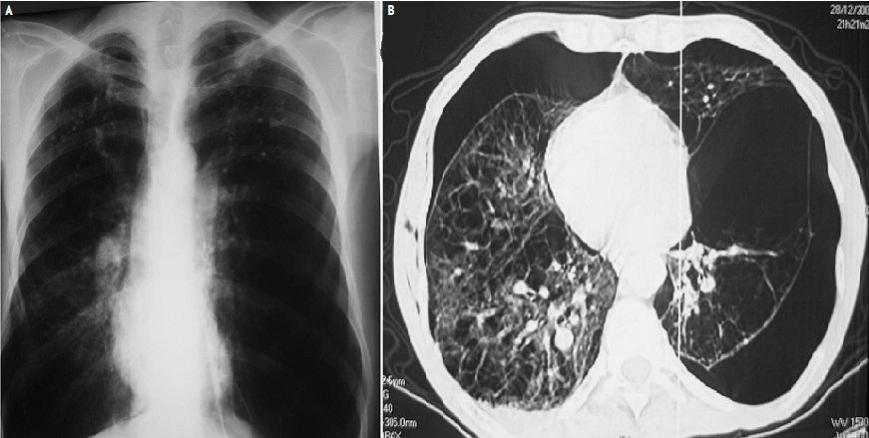
(A) coupe scannographique parenchymateuse montrant le pneumothorax bilatéral sur poumon emphysémateux; (B) coupe scannographique parenchymateuse montrant le pneumothorax bilatéral sur poumon emphysémateux

